# Idiopathic pancreatitis is a consequence of an altering spectrum of bile nucleation time

**DOI:** 10.1186/1756-0500-4-163

**Published:** 2011-05-26

**Authors:** V Abeysuriya, KI Deen, BK Dassanayake, SK Kumarage, NMM Navarathne, A Pathirana

**Affiliations:** 1 Department of Clinical Anatomy, Faculty of Medicine, Ragama, University of Kelaniya, Sri Lanka; 2 Department of Surgery, Faculty of Medicine, Ragama, University of Kelaniya, Sri Lanka; 3 University Department of Surgery, The North Colombo General Hospital, Ragama, Sri Lanka; 4 Department of Surgery, Faculty of Medicine, Ragama, University of Kelaniya, Sri Lanka; 5 National Hospital of Sri Lanka, Colombo, Sri Lanka; 6 Department of Surgery, Faculty of Medical Sciences, University of Sri Jayawardenapura, Sri Lanka

## Abstract

**Background:**

The pathogenesis of idiopathic pancreatitis (IP) remains poorly understood. Our hypothesis is that IP is a sequel of micro-crystallization of hepatic bile.

**Methods:**

A prospective case control study compared 55 patients; symptomatic cholelithiasis - 30 (14 male, median age 36 years; mean BMI - 25.1 kg/m^2^), gallstone pancreatitis - 9 (3 male, median age 35 years; mean BMI - 24.86 kg/m^2 ^) and IP - 16 (9 male, median age 34 years; mean BMI -23.34 kg/m^2^) with 30 controls (15 male, median age 38 years; mean BMI = 24.5 kg/m^2^) undergoing laparotomy for conditions not related to the gall bladder and bile duct. Ultrafiltered bile from the common hepatic duct in patients and controls was incubated in anaerobic conditions and examined by polarized light microscopy to assess bile nucleation time (NT). In the analysis, the mean NT of patients with gallstones and gallstone pancreatitis was taken as a cumulative mean NT for those with established gallstone disease (EGD).

**Results:**

Patients were similar to controls. Mean NT in all groups of patients was significantly shorter than controls (EGD cumulative mean NT, 1.73 +/- 0.2 days vs. controls, 12.74 +/- 0.4 days, P = 0.001 and IP patients mean NT, 3.1 +/- 0.24 days vs. controls, 12.74 +/- 0.4 days, P = 0.001). However, NT in those with IP was longer compared with those with EGD (mean NT in IP, 3.1 +/- 0.24 days vs. cumulative mean in EGD: 1.73 +/- 0.2 days, P = 0.002).

**Conclusion:**

Nucleation time of bile in patients with IP is abnormal and is intermediate to nucleation time of lithogenic bile at one end of the spectrum of lithogenicity and non-lithogenic bile, at the other end.

## Background

Idiopathic pancreatitis (IP) accounts for 8% to 44% of all causes of acute or acute-on-chronic pancreatitis [1-3]. More recently, autoimmune disease has been reported to account for another 5% of patients with idiopathic chronic pancreatitis [4]. Biliary microlithiasis (gallstones < 3 mm) is another known causative factor [1-7]. We have previously shown that hepatic biliary nucleation time is reduced in patients with gallstones compared with controls [7]. This study was designed to evaluate the characteristics of hepatic bile nucleation time in patients with idiopathic acute pancreatitis, gallstone pancreatitis and symptomatic gallstones without pancreatitis. Data were compared with controls without biliary symptoms or established biliary sludge or gallstones.

## Methods

Between March 2006 and April 2009, fifty five patients (symptomatic cholelithiasis-30, gallstone pancreatitis- 9, idiopathic pancreatitis-16 (Table 1) and thirty historic controls were studied. Controls comprised those who underwent laparotomy for abdominal pathology other than for hepatobiliary and pancreatic disease. In all control subjects, the gallbladder and extra-hepatic biliary anatomy was normal on ultrasound and there were no demonstrable gallstones. Informed written consent was obtained from all patients who were recruited to the study. Ethical clearance was granted by the Ethical Committee of the Faculty of the Medicine, Ragama, university of Kelaniya, Sri Lanka

**Table 1:** Characteristics of patients and controls

	Female	Male	Median age (range) in years	BMI +/-(SEM) Kg/m 2	P value
Gallstone pancreatitis (n - 9)	6	3	35 (33-40)	24.86 +/-0.23	P = 0.1(t-test)
	
Idiopathic pancreatitis (n - 16)	7	9	34 (31-39)	23.34 +/-0.2	P = 0.1(t-test)
	
Symptomatic cholelithiasis (n -30)	16	14	36 (33-71)	25.1 +/-0.33	P = 0.1(t-test)
	
Controls (n = 30)	15	15	38 (33-70)	24.5+/-0.23	

### Patients - Inclusion criteria: Established gallstones

Patients who had trans-abdominal ultrasound (US) proven stones in the gallbladder and/or the main bile duct, without clinical, biochemical and radiological evidence of pancreatitis, were considered in the group of patients with gall stones without pancreatitis. The aforementioned group of patients presented to the outpatient department with symptoms of biliary colic. Those who presented to the emergency room with epigastric pain and tenderness, serum amylase > 3 times the normal range, elevated serum alanine transaminase (> 60 IU/l within 48 hrs of presentation), serum C-reactive protein > 150 mg/l up to 72 hours after onset of symptoms, main bile duct dilatation on ultrasonography: > 8 mm diameter with the gallbladder in situ, or > 10 mm following cholecystectomy, comprised those with gallstone pancreatitis [3-6]. A pre-requisite for inclusion in the group with gallstone induced pancreatitis was, in all of these patients, gallstones were found to be present in the gall bladder or the bile ducts by either trans-abdominal ultrasound, magnetic resonance choledocho-pancreatography (MRCP) or at the time of endoscopic retrograde choledocho-pancreatography (ERCP), if indicated.

### Idiopathic pancreatitis

Patients who had had a second or third episode of pancreatitis with serum amylase > 3 times the normal range, elevated serum alanine transaminase (> 60 IU/l within 48 hrs of presentation), serum C-reactive protein > 150 mg/l up to 72 hours after symptoms, where no other etiology of pancreatitis was found in the detailed clinical history, physical examination, haematological, biochemical, non interventional (US, MRCP or computerised tomography) and interventional (ERCP) radiological assessments, were recruited to the study as patients who had idiopathic pancreatitis [5-11]. All of these patients included in the study underwent ERCP examination and it had to be demonstrated that, in all patients in this group, bile obtained at ERCP was devoid of micro-crystals under polarized light microscopy using criteria previously reported [7].

### Patients - Exclusion criteria

Those who had had a single episode of acute pancreatitis, bile containing sludge or micro-crystallisation, patients who did not give consent, those who consumed alcohol or smoked, those taking the oral contraceptive pill and patients with a history of autoimmune diseases and family history of inherited disease were excluded. Furthermore, those with obstructive jaundice and who were too ill to be studied were also excluded from further study.

### Historical controls

30 controls (15 female: 15 male, median age 38 years, range 33-70 years, mean+/-SEM BMI = 24.5+/-0.23 kg/m^2^), who underwent laparotomy for other abdominal pathology; large bowel cancer (22), oesophageal cancer (3), oesophageal stricture (3) and gastric cancer (2) were selected for evaluation of bile nucleation time at the university surgical unit Kelaniya, Sri Lanka. All of the control individuals had normal gallbladder and extra-hepatic biliary anatomy on ultrasound with no demonstrable gallstones [7]. Informed written consent was obtained from all patients who were recruited to the study. Ethical clearance was granted by the Ethical Committee of the Faculty of Medicine, university of Kelaniya, Sri Lanka [7].

### Interventional procedures

All endoscopic procedures were performed under conscious sedation with a side-viewing duodenoscope (TJF160R. Olympus Optical Co. Tokyo, Japan). Patients with gall stone pancreatitis were offered wide sphincterotomy, balloon sweeping of the bile duct and common bile duct stenting with 7 or 10 Fr. stents, whereas those patients with idiopathic pancreatitis were offered wide sphincterotomy, balloon sweeping of the bile duct and stenting of the pancreatic duct with or without stenting of the common bile duct with 7 or 10 Fr stents. All had a single intravenous dose (1.2 mg) of co-amoxiclavulinic acid at the time of the procedure followed by two post-operative doses of antibiotic as prophylaxis.

All surgical procedures were performed under general anaesthesia with intermittent positive pressure ventilation. Those with gallstones underwent laparoscopic cholecystectomy using the standard four-port technique. Those who were control subjects underwent laparotomy through a midline incision. All those who underwent laparoscopic cholecystectomy had a single intravenous dose (1.2 mg) of co-amoxiclavulinic acid at the time of induction of anaesthesia. Control subjects, who underwent laparotomy had a single intravenous dose (1.2 mg) of co-amoxiclavulinic acid and intravenous metronidazole 500 mg at the time of induction of anaesthesia, followed by two post-operative doses of antibiotic as prophylaxis.

### Collection of bile

In patients with gallstone pancreatitis and idiopathic pancreatitis, 2.5 ml of bile was aspirated from the common hepatic duct by using an endoscopic canula (TJF160R. Olympus Optical Co., Tokyo, Japan), mounted on a 10 cc syringe. In those who underwent laparoscopic cholecystectomy, bile was aspirated from the common bile duct through a cholangio-catheter introduced laparoscopically and secured with an endo-clip. During aspiration, the first milliliter of bile was discarded and the subsequent 2.5 ml was aspirated into a sterile container. In control subjects, 2.5 ml of bile was aspirated from the common bile duct by needle puncture using an18 gauge needle mounted on a 10 cc syringe. All bile samples were stored at -70°C until the time of analysis [7].

### Assessment of nucleation time

Stored bile was initially ultra-centrifuged at 100,000 g (25000 rpm) and filtered using a 0.2 μ cellulose nitrate membrane filter (0.2 μ, 25 mm, Whatman Int. Ltd. Maidstone, England). A drop of bile was examined under polarized light microcopy (PLM) to confirm the absence of crystals in the bile samples and bile was incubated at 37°C under anaerobic conditions. Thereafter, bile was examined daily by polarized light microscopy (PLM), for appearance of cholesterol monohydrate crystals by the principal investigator. Nucleation time (NT) of bile was defined as the time taken for the first crystals to appear under PLM. [7,12-17]

### Statistical analysis

Data are expressed as either median (range) or mean (SEM) and analyzed using the Statistical Package for Social Sciences version 11, (SPSS 11.0, Chicago, Illinois, USA). The mean nucleation time in patients with gallstones and gallstone pancreatitis, considered as a group with established gallstones, idiopathic pancreatitis and controls, the possible effect of age and BMI on nucleation time was evaluated using a general linear model, which generated an "F" statistic. To assess which of the three confounders had the most effect (i.e. age, BMI, clinical group), a "type 111 sum of square" test was effected, which also generated an "F" statistic. If there was a significant difference in mean nucleation time between the groups, a Bonferroni mean separation test was employed to assess if the nucleation times of the three groups is significantly different. Also, the mean nucleation time in patients with gallstones and controls was compared using the t-test. Significance was assigned to a p-value of < 0.05.

## Results

**Figure 1:**
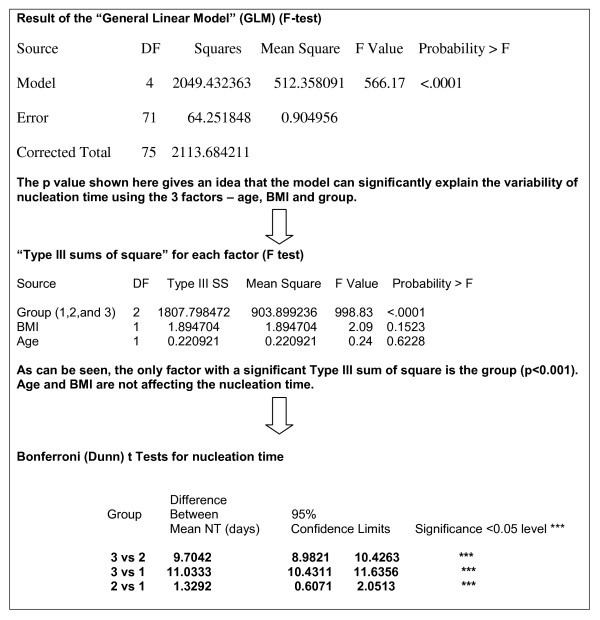
**The flow chart indication the sequence of statistical analysis in evaluation of significance for mean nucleation time in patients with gallstones (group 1), idiopathic pancreatitis (group 2) and control subjects (group 3)**.

All patients underwent successful endotheraputic procedures with biliary cannulation and aspiration of bile without morbidity. There was no procedure related mortality in the first 60 days in any group after operation. Patients were similar to controls in age, gender and BMI (Table 1). We found that the General Linear Model was able to significantly explain the variability of nucleation time using the 3 factors - age, BMI and any of the clinical groups. Thus, three confounding factors were encountered in initial analysis (Figure 1). The "type III sums of square" test, however, revealed that age and BMI did not significantly impact upon the result of nucleation time. The Bonferroni mean separation test, used to test the mean NT of those with established gallstones vs. idiopathic pancreatitis vs. control subjects, showed significant differences between each of the three groups of test subjects (Figure 1). A t-test, which compared mean NT between the control group and patients showed that mean NT was significantly longer in controls (12.74 days) compared with idiopathic pancreatitis (3.1 days), gallstone pancreatitis (1.73 days) and gallstones without pancreatitis (1.73 days - Table 2). There was no difference in the mean NT of bile in those with gallstones with pancreatitis and without pancreatitis (Table 2). Therefore, data for those with gallstones, with and without pancreatitis, were pooled together and expressed as a cumulative mean NT for comparison. Thus, mean NT of hepatic bile in idiopathic pancreatitis subjects was greater than mean NT for subjects with established gall stones (gallstones with and without pancreatitis). In summary, mean nucleation time was longest in the bile of controls. Likewise, nucleation time in idiopathic pancreatitis bile was longer than nucleation for gallstones.

**Table 2:** Mean, standard error of the mean (SEM) and range of bile nucleation times (NT) in controls, idiopathic pancreatitis, gallstone pancreatitis and gallstones without pancreatitis.

Group	Mean +/- (SEM) NT in days	p- value
Controls (n = 30)	12.74 +/- 0.4(11-15 days)	**

Idiopathic pancreatitis (IP) (n = 16)	3.1 +/- 0.24 (2- 6 days)	** **#**

Gallstone pancreatitis (GP) (n = 9)	1.73 +/- 0.2 (1- 4 days)	** **#**

Gallstones without pancreatitis (GWP) (n = 30)	1.73+/-0.2 (1-3 days)	** **#**

NT of establish gallstone disease (EGD = GP + GWP)	1.73/- 0.2 (1-4 days)	**#**

*Significance is assigned to a p-value < 0.05, ** p = 0.001 (t-test), **# **p = 0.002 (t-test)

## Discussion

Pancreatitis is defined by the presence of inflammation in an otherwise normal gland and is diagnosed with the aid of laboratory values and imaging studies. Acute pancreatitis can lead to recurrent acute pancreatitis if the underlying factor remains uncorrected. Usually, acute pancreatitis results from alcohol abuse or gallstone disease. Initial evaluation fails to detect the cause of pancreatitis in 10% to 30% of patient and, as a result, the diagnosis of "idiopathic" pancreatitis is made. In these patients, extensive evaluation, including specialized laboratory investigations, endoscopic retrograde cholangiopancreatography, endoscopic ultrasound, or magnetic resonance cholangiopancreatography, typically leads to a diagnosis of microlithiasis, sphincter of Oddi dysfunction, or pancreas divisum [1-7]. Despite identification of various causative factors, in about 20% of all patients, the aetiology will continue to remain idiopathic [4,13].

Cholesterol crystallization is the initial step in the complex process of gallstone formation and its subsequent complications such as gallstone pancreatitis [1,4,18,19]. Micorcrystals refers to cholesterol monohydrate, calcium bilirubinate, or calcium carbonate [14,15,20]. In most reports, bile aspiration from the duodenum or from the bile duct yields microcrystals in 50% to 73% of patients with idiopathic pancreatitis [21]. A variety of methods for obtaining bile for assessment of nucleation time and microcrystals have been described; nasoduodenal aspiration, nasobiliary aspiration, endoscopic extraction, percutaneous gallbladder puncture and aspiration during operation [22-24]. Duodenal bile is not ideal for determination of nucleation time, due to contamination by pancreatic enzymes. Nasobiliary aspiration of bile may be superior, but collection of bile via a nasobiliary tube after cholecystokinin injection, may carry a risk of pancreatitis [21,22]. Endoscopic cannulation, visualization of the extra-hepatic biliary system and bile aspiration has its own risks of pancreatitis [23,24]. In our study, we chose trans-laparoscopic bile aspiration as our method for obtaining bile samples at the time of laparoscopic cholecystectomy. Also, direct cannulation of the bile duct would avoid the sampling bias of aspirating stagnant bile in the gallbladder as in nasoduodenal or nasobiliary techniques. Ideally bile should have been obtained from healthy individuals, to serve as controls, but this was not possible. True idiopathic pancreatitis is due to microcrystalisation of hepatic bile. This may be different from the established gallstone pancreatitis, where, pancreatitis is thought to result from passage of a gallstone through the common bile duct, causing a degree of ductal obstruction. Gallstone disease, gallstone pancreatitis and idiopathic pancreatitis do not appear isolated clinical entities. Rather, they appear to be manifestations of an altering spectrum of "nucleation time" of lithogenic bile. Idiopathic pancreatitis, which is associated with abnormal bile nucleation time, should be termed, "microcrystal pancreatitis".

Our study has shown that mean nucleation time in all groups of patients (symptomatic cholelithiasis, gallstone pancreatitis and idiopathic pancreatitis) was significantly shorter than control subjects. However, the nucleation time of those with idiopathic pancreatitis, though less than controls, was greater than the nucleation time of bile where gallstones were present (symptomatic cholelithiasis and gallstone pancreatitis). Because there are no established clinical tests to identify individuals with lithogenic bile, which may result in first time presentation with acute pancreatitis, this group of patients remain an unidentified risk for acute pancreatitis and its consequences. The timing of the onset of idiopathic pancreatitis is such that it affects predominantly young people in the prime of their life, as we have seen in this study. To the best of our knowledge, there has been no study which has compared NT in patients with idiopathic pancreatitis and those with gallstones. In the future, studies should attempt to identify clinical risk profiles of patients with "idiopathic pancreatitis" who subsequently test positive for micro-crystallization when bile is obtained at ERCP. Also, what could be done to reverse the lithogenicity of bile remains largely unexplored and is an area that deserves further study.

## Conclusions

Nucleation time in bile in patients with "idiopathic pancreatitis" is abnormal. In these patients, bile nucleation time is intermediate to that of non-lithogenic biliary controls, at one end of the spectrum, and lithogenic bile in those with established gallstones, at the other end of the spectrum.

## Competing interests

The authors declare that they have no competing interests.

## Authors' contributions

All authors read and approved the final manuscript. VA wrote the main body of the article under supervision of KID. BD helped with statistical evaluation. SKK, NMMN, AP provided advise on clinical aspects. VA is the guarantor.

## Sources of financial support

Self
